# From basin to gulf: Conservation tillage improves soil health but exacerbates hypoxia

**DOI:** 10.1038/s44264-025-00090-0

**Published:** 2025-08-28

**Authors:** Kang Liang, Xuesong Zhang, Gregory W. McCarty, Kaiguang Zhao, Feng Gao

**Affiliations:** 1 Oak Ridge Institute for Science and Education, Oak Ridge, TN USA; 2 USDA-ARS Hydrology and Remote Sensing Laboratory, Beltsville, MD USA; 3 School of Natural Resource Sciences, North Dakota State University, Fargo, ND USA; 4 School of Environment and Natural Resources, Ohio Agricultural and Research Development Center, The Ohio State University, Wooster, OH USA

**Keywords:** Carbon cycle, Environmental sciences

## Abstract

Agricultural management practices such as conservation tillage is promoted in the U.S. Midwest for improving soil health, mitigating nutrient loss, and reducing hypoxia in the Gulf of America (GOA). However, large-scale evaluations of tillage impact on soil organic carbon (SOC), water quality, and the implications for hypoxia in the Gulf are lacking. By combining a meta-analysis of field experiments with watershed modelling, this study finds that by 2050, no-till (NT) farming could enhance SOC by ~5.4 MgC ha^−^^1^, increase streamflow by 17.3%, and reduce soil erosion by ~4.9%, compared to high-intensity tillage (HT). However, widespread NT adoption could raise nitrogen loss, thus expand summer hypoxia of the GOA to 16,500 km², 21.5% larger than the HT scenario. Despite its soil health benefits, conservation tillage may complicate efforts to reduce hypoxic zones to the targeted 5000 km² by 2035. These tradeoffs underscore the need for balanced approaches in future conservation strategies.

## Introduction

Excessive nutrient discharge into coastal systems is a primary driver of the globally widespread hypoxic zones—areas of oxygen depletion—in coastal waters, posing a severe threat to marine ecosystems^1^. Agricultural runoff enriched with nutrients from the Mississippi River Basin (MRB), especially from the agriculturally intensive U.S. Midwest, contributes to the persistent hypoxic zones in the Gulf of America (GOA). These oxygen-deficient zones have severe environmental and socio-economic impacts, jeopardizing the $2.8 billion yr^−1^^1^ commercial and recreational fishery industries in the northern GOA^2–5^. In response, the U.S. government has set a goal of reducing the GOA hypoxic zone to 5000 km^2^ by 2035^6^, which requires at least a 45–60% reduction in nitrogen loading from the MRB^5,7^.

Beyond environmental concerns, elevated nitrate levels in drinking water pose serious health risks, including cancer and adverse birth outcomes, with associated medical and indirect costs in the U.S. reaching up to $8 billion annually^8,9^. Despite decades of conservation efforts in the U.S. Midwest, neither nitrogen loading to the GOA nor the extent of the hypoxic zone in the region has declined.

**Fig. 1:**
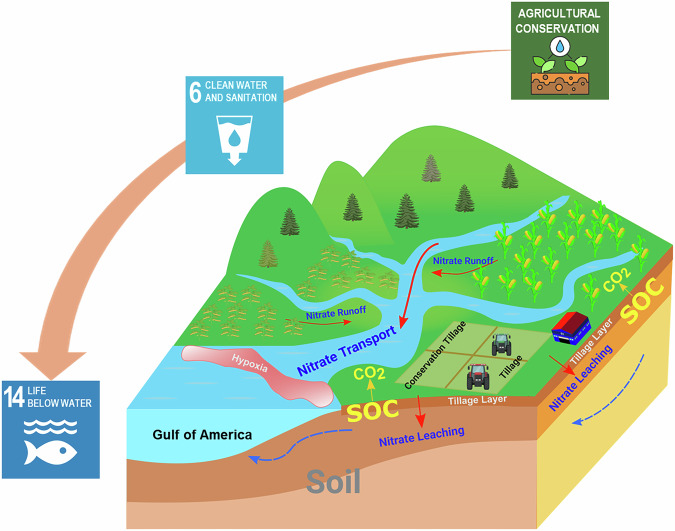
Schematic illustration of the impacts of conservation tillage on soil health, water quality, and hypoxia in coastal waters. Actions in the upstream watershed have far-reaching and multi-dimensional impacts, contributing to initiatives such as the United Nations Sustainable Development Goals (e.g., SDG 6: Clean Water and Sanitation and SDG 14: Life Below Water).

Nearly half of the nitrogen discharged into the GOA originates from the Upper Mississippi River Basin (UMRB), despite the region comprising only 15% of the total MRB area^10^. With ~50% cropland coverage, the UMRB has become a hotspot for implementing agricultural management practices, such as conservation tillage, to reduce nitrogen runoff into coastal waters^11^. In both the U.S. Midwest and globally, conservation tillage practices—including no-till (NT) and reduced tillage—have been increasingly promoted for their potential to enhance soil organic carbon (SOC) accrual, mitigate soil erosion, and preserve soil moisture (Fig. 1)^12,13^. Currently, conservation tillage is adopted on 76% of U.S. corn acreage, 74% of soybean acreage, and 68% of wheat acreage. Notably, the adoption of NT practices for wheat and corn has nearly doubled over the past two decades^14^, now covering 47% of corn, 53% of soybean, and 66% of wheat cultivation.

The environmental consequences of NT practices are multidimensional and complex, with both demonstrated benefits and ongoing uncertainties^15,16^. Extensive research has demonstrated the potential of NT adoption to enhance SOC accumulation, though its effectiveness varies by region and cropping system^17^. However, the influence of conservation tillage on water quality—particularly nitrate leaching—remains highly debated. A recent global meta-analysis suggests that shifting from conventional tillage to NT may reduce nitrate leaching by 19.5%^18^. In contrast, other studies indicate that such a transition could lead to increased nitrate leaching^19,20^. These conflicting findings underscore the need for a comprehensive assessment of how the widespread adoption of conservation tillage in the U.S. Midwest affects soil health and nutrient loss, particularly its implications for hypoxia in coastal waters.

This study investigates the trade-offs associated with agricultural conservation tillage practice, aiming to mitigate unintended negative consequences and support the long-term success of agricultural conservation efforts^21^. First, we conducted a meta-analysis of field data to assess the effects of conservation tillage on SOC accrual and nitrate leaching in corn-soybean production systems across the MRB and surrounding regions. Next, we applied watershed modeling to estimate the long-term impacts of large-scale NT adoption on SOC and nitrate load. Historical data were then analyzed to establish the relationship between nitrate loading from the UMRB and the extent of hypoxia in GOA. Finally, we explored the synergies and trade-offs of conservation tillage in addressing soil health and water quality challenges. Our findings provide new insights into the role of conservation tillage practices in achieving the U.S. government’s goal of reducing the GOA hypoxic zone to 5000 km² by 2035, while ensuring that agricultural conservation efforts avoid unintended adverse consequences.

## Results

### Meta-analysis of tillage effects on soil carbon and nitrate leaching

**Fig. 2:**
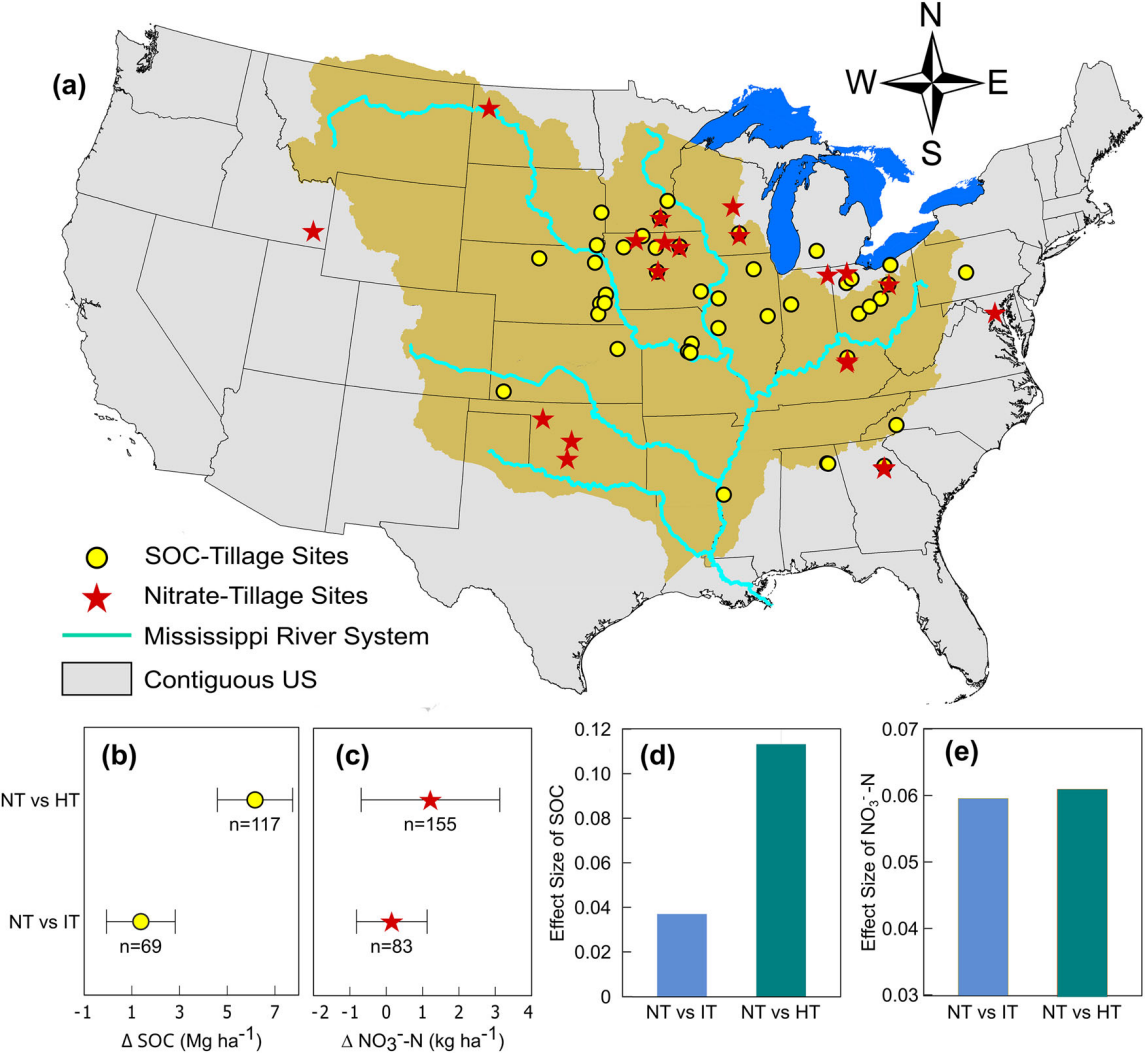
Soil organic carbon and nitrate leaching effects of different tillage practices: no-till (NT), intermediate tillage (IT), and high-intensity tillage (HT). **a** Spatial distribution of field studies included in the meta-analysis; **b** Effects of tillage practices on SOC at a depth of 0–30 cm; **c** effects of tillage practices on nitrate leaching; **d** Effect size of tillage impacts on SOC; **e** Effect size of tillage impacts on nitrate leaching (NO3- - N). NT-IT and NT-HT refer to the difference between NT and IT, and NT and HT, respectively. Colored dots represent the mean difference, with error bars representing the 95% confidence interval. The numbers below the dots refer to the number of paired observations.

Our synthesis of 1071 field observations revealed that NT could significantly increase SOC in the top 30 cm of soil in corn production systems across the MRB and surrounding regions (Fig. 2A & B). Based on paired observations and compared to high-intensity tillage (HT), NT on average increased SOC stocks from 50.8 MgC ha^−1^ to 57.0 MgC ha^−1^ in the top 30 cm — a 14.2% increase. Compared to intermediate tillage (IT), NT increased SOC stocks from 58.6 MgC ha^−1^ to 59.9 MgC ha^−1^—a 4.7% increase (Fig. 2B). Similarly, nitrate leaching under NT was significantly higher, exceeding that under HT and IT by —4.9% and 0.6%, respectively (Fig. 2A & C). The estimated effect sizes were 0.11 (NT vs. HT) and 0.04 (NT vs. IT) for SOC, and ~0.06 (NT vs. IT or NT vs. HT) for nitrate leaching (Fig. 2D & E), suggesting that NT adoption leads to measurable increases in both SOC and nitrate leaching.

### Multi-dimensional impacts of tillage practices

**Fig. 3:**
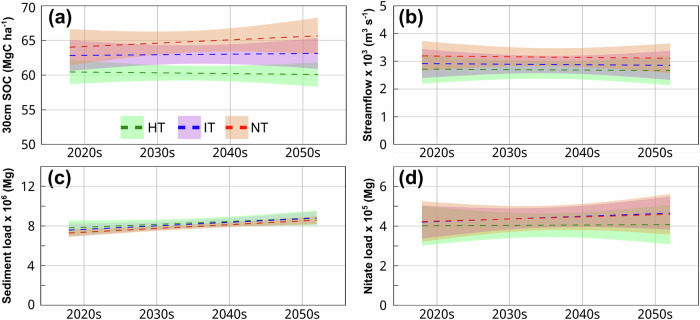
Environmental impacts of tillage practices in the UMRB from the 2020s to 2050s. **a** Top 30 cm SOC, **b** streamflow, **c** sediment load, and **d** nitrate load for high-intensity tillage (HT), intermediate-intensity tillage (IT), and no-till (NT) practices. The dashed line represents the trendline. The shaded area indicates the standard deviation.

The observed effects of NT on SOC and nitrate leaching from the meta-analysis were further corroborated by independent watershed modeling (Fig. 3). Using the Soil and Water Assessment Tool (SWAT)—a widely endorsed watershed modeling tool —we simulated the impacts of the wide adoption of three tillage scenarios (NT, IT, and HT) for corn-soybean production systems in the UMRB^22,23^. The model demonstrated strong predictive performance for simulating streamflow, nitrate load, total nitrogen load, sediment load, and SOC when evaluated against field observations (see Supplementary Fig. 1 & Fig. 2). These simulations addressed the spatial and temporal limitations of field-based analysis, allowing us to assess large-scale impacts of tillage adoption while providing mechanistic insights through multiple environmental metrics.

Consistent with meta-analysis, the watershed model showed that NT adoption increased SOC. Of the three tillage scenarios, HT resulted in the largest SOC loss (Fig. 3A). By 2050, SOC levels in the top 30 cm of soil under NT were projected to be 5.4 MgC ha^−1^ (9.1%) and 2.2 MgC ha^−^^1^ (3.5%) higher than under HT and IT, respectively, comparable in both magnitude and direction to the meta-analysis results. The SOC impact was particularly concentrated in the top 30 cm of the soil profile. About 88.5% of the SOC difference between NT and HT, and 91.7% between NT and IT, was attributable to the top 30 cm layer (Fig. 3A).

The watershed model also showed a pronounced increase in nitrogen pollution when shifting from HT or IT to NT practices (Fig. 3D), reinforcing the meta-analysis results. During 2020–2050, annual nitrate loading under NT was projected to be 10.8% and 1.5% higher than that of HT and IT, respectively. This elevated loss resulted from multiple interacting factors, with the dominant one being higher water yields with NT, which promotes the movement of nitrate through the soil profile into waterways. While NT adoption reduced annual sediment loads from the UMRB by 4.9% (compared to HT) and 3.2% (compared to IT)—a factor that could help mitigate nitrogen inputs to streams (Fig. 3C), this benefit was outweighed by higher streamflow under NT (Fig. 3B).

During the same period, the average annual streamflow under NT was 17.3% and 9.4% higher than under HT and IT, respectively. Therefore, despite the benefits of NT in improving soil health, water availability, and soil protection, its higher water yield would facilitate nitrate transport through the soil profile and into waterways and contribute to greater nitrate losses.

### Linkages between nitrate loss from UMRB and hypoxia in coastal waters

**Fig. 4:**
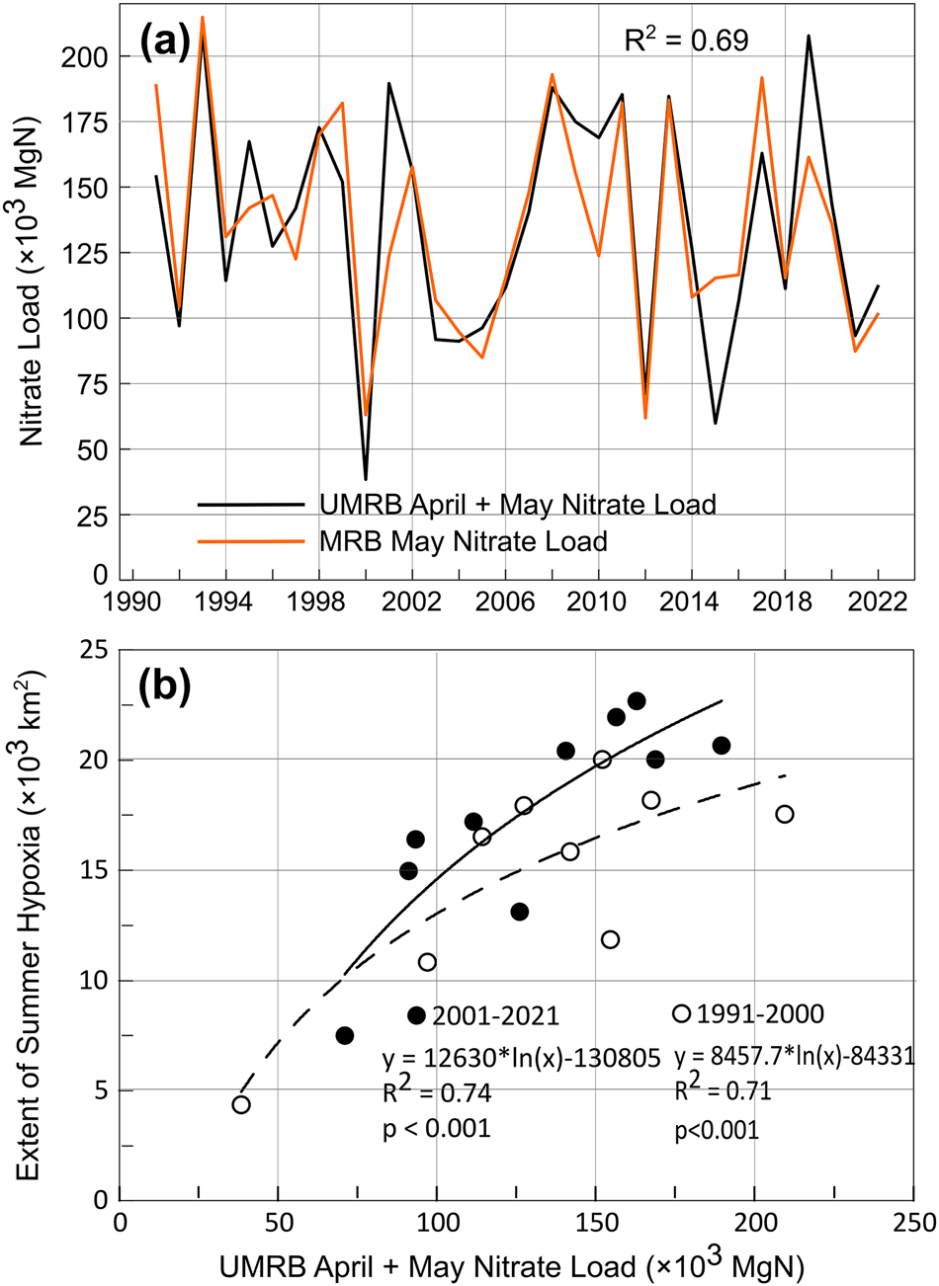
Relationship between April+May nitrate load of the UMRB and May nitrate load of the MRB and the size of the hypoxic zone in the GOA. **a** Correlation between April+May nitrate load of the UMRB and May nitrate load of the MRB. **b** Relationship between April+May nitrate load of the UMRB and the size of the hypoxic zone of coastal waters near the Mississippi River delta.

We analyzed historical nitrate load data from river gauges of the UMRB and the MRB alongside cruise-based observations of hypoxic zones in late mid-summer (typically the last week of July) in the GOA for 1991–2022. The data revealed a strong correlation between the April+May nitrate load of the UMRB and the May nitrate load of the MRB (Fig. 4A, R^2^ = 0.69). Additionally, the UMRB April+May nitrate load was found to be strongly positively correlated with the extent of summer hypoxia in the GOA (Fig. 4B). Between 1991 and 2022, the average April+May nitrate load from the UMRB was 135,907 MgN, while the average May nitrate load from the MRB over the same period was 135,316 MgN.

Given the high correlation between the UMRB’s April+May nitrate load and the MRB’s May nitrate load (Fig. 4A), we used the April+May UMRB nitrate loads as a surrogate for the May MRB nitrate load in the LSU hypoxia model^24–26^ that we chose to predict the size of summer hypoxic zones in the GOA. The revised model explains 74% and 71% of the observed variabilities of the extent of summer hypoxic zone in the GOA for the periods 1991–2000 and 2001–2021, respectively (Fig. 4B).

### Implications of conservation tillage in the UMRB for the hypoxia in the Gulf of America

**Fig. 5:**
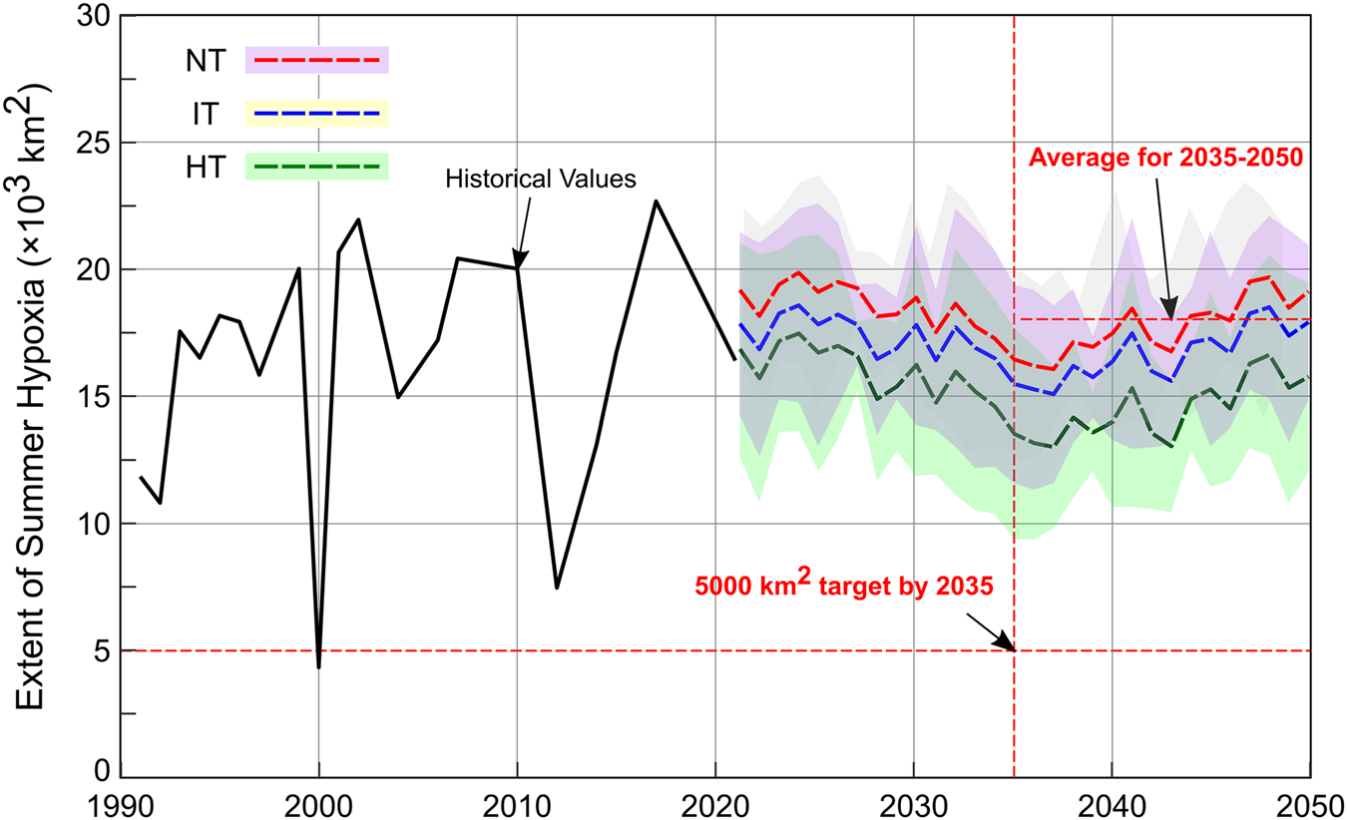
Historical and projected hypoxic zone in the Gulf of America. The 5-year moving average of the extent of the hypoxic zone in the GOA. Data prior to 2021 are observed values; estimates from 2021 onward are driven by climate data from GCMs. The shaded area indicates the standard deviation.

We integrated the watershed model simulation of April-May nitrate load for UMRB into the LSU hypoxia model to predict the extent of GOA hypoxic zones for the period of 2021 to 2050 (Fig. 5). The predictions indicated that large-scale adoption of NT could significantly expand hypoxic zones in the GOA compared to IT or HT. By 2035, the estimated 5-year average of the hypoxic zone extent would reach approximately 16,500 km² under NT, which is substantially larger than the sizes under IT (15,500 km^2^) and HT (13,600 km²). Between 2035 and 2050, the projected hypoxia extents would further increase, reaching up to 17,800 km², 16,700 km², and 14,500 km² for NT, IT, and HT practices, respectively.

The persistence and expansion of the hypoxic zone result from the complex interplay between the hydrological cycle, agricultural management practices, and nitrogen loss. For example, NT practices can increase runoff and streamflow, which in turn enhances nutrient transport and exacerbates hypoxic conditions in coastal waters. The Hypoxia Task Force (2022) emphasized that nutrient delivery to the Gulf—and consequently, the severity of hypoxia—is heavily influenced by water discharge from the MRB, as higher streamflow carries greater nutrient loads, fueling the growth of hypoxic zones^5^.

## Discussion

Through integrating a metal-analysis of field observations with watershed modeling, our study comprehensively explores the multi-dimensional effects of conservation tillage practices on soil health and nitrogen loss in the agriculturally intensive UMRB, and the potential impacts on hypoxia in coastal waters. While NT practices enhance soil health and water availability, they come with critical tradeoffs, including exacerbating nitrogen pollution and worsening hypoxia in the GOA, which poses risks to public health and fishery industries. Our findings highlight the need for a nuanced approach to conservation tillage practices, balancing the ecological benefits with their broader environmental consequences.

The results suggest that NT practices substantially enhance SOC accumulation in the topsoil. The meta-analysis revealed a significant increase in SOC stocks by approximately 6.4 MgC ha^−1^ under NT compared to HT, with effect sizes of 0.11 and 0.04 for NT vs. HT and NT vs. IT, respectively. These outcomes align with previous studies^16,17,27,28^. For instance, Luo et al.^27^ demonstrated that NT enhances SOC storage in the upper soil layers by reducing soil disturbance, which limits the oxidation and decomposition of organic carbon. Similarly, Haddaway et al.^28^ reported that NT significantly increases SOC concentration in the topsoil, particularly in regions where intensive tillage previously led to substantial carbon loss. However, while NT is effective in promoting SOC accumulation near the surface, Luo et al.^27^ suggested it may result in lower carbon accrual at greater depths due to reduced soil mixing and root penetration, which are more prevalent in HT systems. This stratification of SOC, where most carbon is concentrated near the surface under NT, has been widely reported. For example, both Haddaway et al.^28^ and Crystal-Ornelas et al.^16^ noted that while NT is highly effective in maintaining surface SOC, its ability to enhance soil carbon in deeper soils is less certain and depends on factors such as soil type, climate, and cropping systems.

Compared to HT, NT practices provide significant protection against soil erosion, reducing sediment loss by 6.0% by 2050. The HT practices typically involve frequent inversion of soil and leave the soil surface with less residue, leading to increased soil erosion and sediment transport. This is supported by numerous previous studies that highlight the high erosion potential of conventional tillage methods, particularly those involving deep plowing and frequent soil disturbance^29,30^. In contrast, NT practices can minimize soil disturbance and maintain crop residues on the surface, which helps shield the soil from erosion. This approach significantly reduces the detachment of soil particles, thereby lowering sediment transport by runoff. The simulated reduction in sediment loads under NT supports the broader understanding that conservation tillage practices, including NT, are effective at protecting soil from erosion by preserving soil surface cover^29^.

Our meta-analysis corroborates watershed modeling, demonstrating that NT systems often lead to higher nitrate leaching due to increased drainage. Nitrate leaching is a primary pathway for nitrogen loss in crop production systems^20^. While NT leaves more crop residues on the soil surface, which acts as mulch to lower soil evaporation. This mulch effect allows more water to be retained in the soil and increases percolation, which subsequently enhances streamflow^31^. During 2020–2050, the average annual total nitrogen load under NT is 10.8% and 1.5% higher than that of HT and IT, respectively. This increase in nitrogen loss is primarily due to the higher water yield associated with NT, which facilitates the movement of nitrate through the soil profile and into waterways. These results are consistent with previous field studies showing that NT can improve drainage rates compared to tilled systems. For example, Bakhsh and Kanwar^32^ found that subsurface tile drainage under NT was nearly double that of IT (chisel plow) between 1993 and 1998 in Nashua, Iowa. Similarly, Williams and Robertson^33^ found that NT systems generally retain more water at various soil depths than inversion tillage systems (e.g., moldboard plow), increasing water availability during dry periods. Daryanto et al.^20^ emphasized the importance of pairing NT with complementary practices to enhance nitrogen retention and improve water quality outcomes. Similarly, Li et al.^19^ found that nitrate leaching under NT was significantly higher than HT, though comparable to IT methods. In contrast, other studies, such as Gu et al.^18^ reported reduced nitrate leaching under NT, underscoring the variability of outcomes based on regional soil characteristics, climate conditions, and management practices. These inconsistencies highlight the need for context-specific strategies that maximize the benefits of NT while mitigating its potential drawbacks, such as increased nitrate runoff. The role of key factors such as SOC content, soil texture, duration of NT practice, irrigation, and nitrogen input cannot be overstated. For instance, Li et al.^19^ noted that low SOC content, medium soil texture, short duration, and irrigation conditions heighten the risk of nitrate leaching under NT. These findings emphasize the complexity and multifaceted nature of nitrogen loss dynamics, reinforcing the need for tailored, site-specific approaches to improve nitrate retention and minimize environmental impacts.

Excess nitrate from the UMRB is transported downstream to the Mississippi River and eventually to the GOA, fueling eutrophication and hypoxia. Our results show a strong correlation (R² = 0.69) between the April+May nitrate load from the UMRB and the May nitrate load of the MRB, as well as the extent of hypoxic zones in the GOA. Notably, hypoxic zones have expanded in size and severity over time, suggesting an increased sensitivity of coastal systems to nutrient inputs. This aligns with findings from Turner et al.^25^, who attribute this trend to accumulated organic matter in sediments and heightened sediment oxygen demand.

The projected increase in GOA hypoxia under NT adoption poses significant challenges to achieving the target of reducing hypoxic zones to 5000 km² by 2035. Without aggressive nitrogen load reduction and adaptive management strategies, such as cover crops, reduced nitrogen application rates, and split nitrogen applications, NT could exacerbate nutrient pollution and hinder the progress. These strategies have shown promise in improving soil nitrogen retention and mitigating the negative impacts of NT on water quality, as noted in previous studies^20,34^.

Our findings underscore the need for a holistic approach to conservation tillage practices. The integration of NT with complementary practices can help balance soil health benefits with reductions in nitrate runoff. Additionally, expanded field experiments and large-scale modeling efforts are crucial to developing and accessing solutions that address the multi-dimensional impacts of tillage practices, supporting both sustainable agricultural production, clean water, and healthy coastal ecosystems.

## Methods

### Impacts of tillage intensity on soil health and nitrate leaching

To examine the effects of tillage on SOC and nitrate leaching in corn-soybean cropping systems in the MRB and surrounding regions, we conducted a meta-analysis of field studies published between 1990 and 2024. The literature collection was performed by using Web of Science and Google Scholar. The keywords (“till” or “no till”) and (“SOC” or “soil organic carbon” or “soil carbon” or “soil organic matter”) were used for the initial literature search. Studies were selected based on the following criteria: (1) a comparison of no-till (NT) with either intermediate tillage (IT) or high-intensity tillage (HT); (2) inclusion of corn or soybean in the cropping system; (3) the research location within the MRB or adjacent areas; (4) SOC measurements taken to a minimum depth of 30 cm. This initial search identified 45 studies related to SOC that met these criteria. For all studies, metadata were collected, including citation details, study location (site, latitude, and longitude), crop rotation, crop types, treatment description (e.g., tillage, fertilization, cover crops, etc.), start and end year of treatment), sampling depths, soil type, bulk density, SOC content, etc.

Due to the broad and varied definitions of conventional and conservation tillage practices across different studies, we standardized the classification into three categories: NT, IT, and HT, following Haddaway, et al.^28^. In this classification, NT was defined as no or very minimal tillage (depth <5 cm), IT as non-inversion tillage practices at depths up to 20 cm depth (e.g., one pass with disk or chisel plow), and HT refers to the application of multiple tillage practices that invert the soil profile (e.g., the combination of moldboard plowing with several disk tillage, chisel tillage or subsoiling). Studies that lacked sufficient details on tillage practices or SOC measurements (0–30 cm) for NT vs HT or NT vs IT were excluded from further analysis. Based on these criteria, we used 31 experimental studies in the meta-analysis of the impacts of tillage practices on SOC in corn-soybean production systems. As a result, we compiled 1,071 SOC observations from various depths within the top 30 cm from comparative studies (i.e., NT vs. IT and NT vs. HT) in corn production systems across the MRB and surrounding regions (Fig. 2A). For further analysis, we summarized the data by NT-HT and NT-IT comparisons for the 0–30 cm layer, which led to 117 and 69 paired SOC data points for NT-HT and NT-IT comparisons, respectively. A list of studies included in the meta-analysis of SOC is available in Supplementary Table 1.

To assess nitrate leaching, we reviewed field studies that reported the load of nitrate-N from paired NT vs. IT or NT vs. HT experiments. The keywords (“till” or “no till”) and (“nitrate” or “nitrogen” or “nitrate leaching” or “nitrogen leaching”) were used for the initial literature search. The criteria used for screening nitrogen leaching were: (1) inclusion of comparison between NT and tillage (IT or HT) systems; (2) reporting of at least two years of nitrogen data (leaching or runoff); (3) inclusion of corn or soybean in the cropping system. Given the challenges of collecting soil leachate or runoff, fewer experimental studies on nitrogen leaching or runoff were available compared to SOC. As a result, we identified 26 studies for the meta-analysis of tillage impacts on nitrogen leaching and runoff, including a total of 429 observations, with 155 pairs of NT vs IT, and 83 pairs of NT vs HT data. The literature base for the meta-analysis of nitrate leaching can be found in Supplementary Table 1.

We used a continuous random-effects meta-analysis approach to assess the impacts of NT, IT, and HT on SOC and nitrate loss^35^. Specifically, we utilized a natural log-transformed response ratio (lnRR, Eq. 1) as the effect size to calculate the effects of NT, IT, and HT on SOC accrual and nitrate leaching^36^.

1 $$\mathrm{ln}{RR}=\mathrm{ln}\left(\frac{{X}_{{NT}}}{{X}_{T}}\right)=\mathrm{ln}\left({X}_{{NT}}\right)-\mathrm{ln}({X}_{T})$$

where the subscript of XNT and XT are the mean values of SOC accrual or nitrate leaching under NT and tillage (HT or IT), respectively. A lnRR value of zero indicates no impact, while positive or negative lnRR values suggest NT practices increase or decrease SOC accrual or nitrate leaching, respectively.

### Upper Mississippi River Basin as a major contributor to hypoxia in coastal waters

**Fig. 6:**
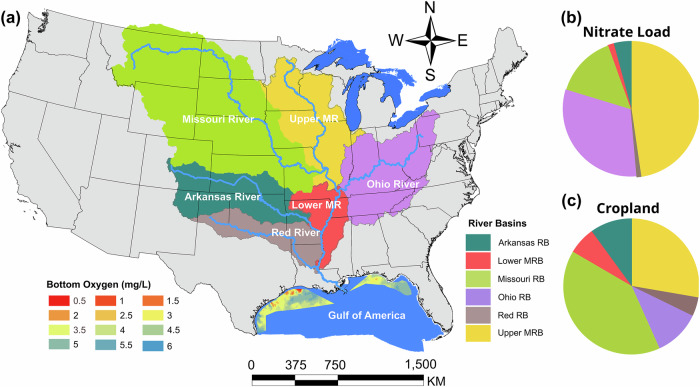
Location of the Upper Mississippi River Basin and its significant contribution to nitrogen pollution and hypoxia in coastal waters. **a** Location of major river basins within the Mississippi River Basin. **b** Contribution of nitrogen load to coastal waters by major basins. **c** Proportion of cropland of major river basins within the Mississippi River Basin. The Bottom Oxygen map on Fig. 6a was based on the survey area from 2017, where values < 2.0 mg/L indicate oxygen-depleted hypoxic zones.

The UMRB, located in the agriculturally intensive U.S. Midwest, is the largest contributor of nitrogen discharged from the MRB to the GOA (Fig. 6). It stretches across multiple states, including Minnesota, South Dakota, Wisconsin, Michigan, Illinois, Iowa, Missouri, and Indiana, and drains an area of 491,702 km^2^. The fertile mollisol soil formed from glacial till and loess deposits makes the region highly productive for intensive agriculture^37^. Corn and soybeans are the dominant crops in the UMRB. From 2006 to 2022, 46.9% of the UMRB’s land was used for agricultural production, predominantly for corn and soybean cultivation, with corn occupying 31.4% and soybean 14.5%. The UMRB has a continental climate, with an average annual precipitation of approximately 900 mm, 75% of which falls between April and October. Although the UMRB comprises only 15.3% of the total area and 27.7% of the cropland in the MRB, it contributes nearly 50% of the MRB nitrogen discharged to the GOA. This makes the UMRB a critical region for the implementation of effective nutrient management strategies to alleviate the detrimental hypoxia in coastal waters^5^.

The primary source of nitrogen pollution in the UMRB is fertilizer use in agricultural production^10^. The Hypoxia Task Force (HTF)’s 2019 and 2021 Report to Congress recommends several agricultural conservation practices to reduce MRB nitrogen inputs to the Gulf^5^. These practices include conservation tillage and no-till farming, which minimize soil disturbance, help retain nutrients, and reduce erosion. Optimal fertilizer management through the 4R framework (Right Source, Right Rate, Right Time, Right Place) is also encouraged to decrease nitrogen loss through runoff. Additionally, cover cropping is recommended to limit nitrogen leaching. These on-farm practices, when combined with other measures such as wetland restoration and riparian buffers, and drainage water management, work collectively to mitigate nutrient losses and improve water quality in the Gulf^5^.

### Hypoxia in the Gulf of America

Hypoxia in the northern GOA primarily occurs in the nearshore coastal waters adjacent to the mouth of the Mississippi River (Fig. 6). This phenomenon is mainly driven by the influx of freshwater and solar heating in the region, which together slows water-column mixing, thereby limiting the exchange of oxygen between surface and deeper waters^3^. Such physical conditions combined with other biogeochemical and physical processes create favorable environments for low oxygen levels, resulting in oxygen-depleted areas (i.e., dissolved oxygen concentration <2 mg/L), known as hypoxic or dead zones. The hypoxic zones in these coastal waters typically extend westward from the mouths of the Mississippi and Atchafalaya rivers and often merge into a continuous stretch that can span 500–600 km from the Mississippi River delta past the Texas border. These hypoxic zones are generally 30–60 km wide and are about 10 m thick. The largest recorded hypoxic zone reached 22,668 km^2^ in 2017^3,38^.

The extensive hypoxic zones in coastal waters pose a significant threat to aquatic life, including fish, shellfish, and corals (https://www.epa.gov/ms-htf). It has severe economic and social consequences, with the fishing industry being particularly affected. Hypoxia reduces critical habitats for commercially valuable species, leading to disruptions in marine ecosystems and fisheries^39^. The Gulf region supports both commercial and recreational fisheries valued at over $2.8 billion annually and has experienced shifts in catch patterns due to hypoxic conditions. For instance, hypoxic conditions force shrimp to migrate, reducing the availability of larger, more valuable shrimp, and resulting in economic losses as smaller, less profitable shrimp dominate the catch^40^. Similar effects are seen in other fisheries, underscoring the importance of management strategies to mitigate the socioeconomic consequences of hypoxia and protect the livelihoods of those dependent on the region’s marine resources. As such, the HTF was established in 1997 to address the causes and effects of eutrophication in the coastal waters near the Mississippi River delta and coordinate resources across federal/state/tribal agencies to reduce the hypoxic zone to 5000 km^2^^41^. Despite extensive efforts, the extent of the hypoxic zone remains well above the target as shown in Figs. 4 and 5. Hypoxia data between 1985 and 2021 is obtained from the Gulf of America Hypoxia website (https://www.gulfhypoxia.net/). Details are available in Supplementary Information.

### Watershed modeling of tillage impacts on soil health and nitrogen loading

We used the Soil and Water Assessment Tool (SWAT)^22^ model to investigate the effects of conservation tillage on SOC and nitrogen loading from the UMRB. Additionally, we assessed how these changes may influence the extent of the hypoxic zone in the GOA. The SWAT model is a well-documented, process-based watershed model designed to simulate water quantity and quality as affected by changes in land use, land management, and climate. It is a widely adopted agri-environmental watershed model^23^ and has been used to support the initiatives of the HTF^5,42^. Recently, the SWAT-C model (recently renamed to Terrestrial and Aquatic Sciences Convergence - TASC)^43,44^ was developed to enable simultaneous simulation of streamflow, nitrogen cycling, and soil organic matter (SOM) dynamics by integrating energy-balanced based freeze-thaw cycle algorithms^45^, soil nitrogen nitrification and denitrification algorithms, and soil carbon algorithms from the CENTURY^46^ and EPIC^47^.

### Conservation management practices

We configured the SWAT model in the UMRB for three different tillage scenarios: NT, IT, and HT, based on the definitions provided by Haddaway, et al.^28^. Note that we assumed NT, IT, or HT was adopted for all corn and soybean crop rotations in each of the above three scenarios due to the lack of long-term, continuous, and spatially explicit information about the adoption of different tillage practices. Consequently, this study aims to demonstrate the potential upper end of the environmental impacts of transitioning from conventional to conservation tillage.

### Climate data

To assess the effects of tillage intensity on nitrate loads from the UMRB, we used climate data from five global circulation models (GCMs) within the Coupled Model Intercomparison Project Phase 6 (CMIP6). The GCMs include GFDL-ESM4 (Geophysical Fluid Dynamics Laboratory Earth System Model version 4), MPI-ESM1-2-HR (Max Planck Institute for Meteorology - Earth System Model version 1.2), MRI-ESM2-0 (Meteorological Research Institute - Earth System Model version 2.0), EC-Earth3 (EC-Earth3 Earth System Model), and CanESM5 (Canadian Earth System Model version 5). We used downscaled data for the above GCMs under the Shared Socioeconomic Pathways (SSPs) 585^48^ or “business-as-usual” scenario from the NASA Earth Exchange Global Daily Downscaled Projections (NEX-GDDP-CMIP6) dataset (https://www.nccs.nasa.gov/services/data-collections/land-based-products/nex-gddp-cmip6).

### Streamflow and water quality data

Streamflow, nitrate, and total nitrogen concentration data at the outlets of major watersheds within the Mississippi River Basin were obtained from various USGS stations. These include the USGS station #5587450 for the UMRB, the #3612500 station for the Ohio River Basin, the #6934500 station for the Missouri River Basin, the #7263620 station for the Arkansas River Basin, the #7381490 station for the Red River Basin, and the #7374000 station for the outlet of the Mississippi River Basin. Monthly nitrate and total nitrogen load were estimated using the Load Estimator (LOADEST) model^49^, LOADEST applies multiple equations for estimating daily constituent load based on daily constituent concentrations and streamflow. The results from the best-performing regression model were used and aggregated to monthly time steps.

The hypoxic zone data between 1985 and 2021 were obtained from the Gulf of Mexico Hypoxia website (https://www.gulfhypoxia.net/). The Hypoxia Research Team measured the size of hypoxic zones in late mid-summer (last week of July typically) through over 7–12 day cruises that map the area. HTF used these measurements to track progress towards the 2035 goal of reducing the extent of the hypoxic zone to 5000 km^2^. No value was available for 1989 (no funding available) and 2016. Data for the following 10 years were excluded from the analysis because there were strong storms or unusual wind conditions just before or during the measurement: 1998, 2003, 2005, 2008, 2010, 2011, 2013, 2018, 2019, 2020. Salient examples of hurricanes include Hurricane Barry in 2019 and Hurricane Hanna in 2020. These determinations were confirmed by review of the US National Weather Service records (http://www.nhc.noaa.gov/archive/storm_wallets/atlantic/). These storms disrupted the water column, leading to re-aeration Hypoxia Task Force^5,26^, as confirmed by comparison of pre and post-cruise sampling data collected during the cruise.

### SOC data for model evaluation

We compiled 1219 SOC measurements at various depths, ranging from 5 cm to 120 cm, from seven experimental sites across the U.S. Corn Belt for our model evaluation Liang, et al.^44^. Tillage practices were classified into three categories: no-till (NT), intermediate-intensity tillage (IT, e.g., chisel/disk plow), and high-intensity tillage (HT, e.g., moldboard plow), based on the criteria established for the meta-analysis by Haddaway, et al.^28^. Additionally, SOC measurements were grouped into two depth categories: the top 30 cm and below 30 cm.

Detailed descriptions of the experimental designs for the selected sites are available in the following references: Clay, et al.^50^ for Aurora, SD, Del Grosso, et al.^51^ for Ames, Iowa, GLBRC for Arlington, WI; Nafziger and Dunker^52^ for Champaign, IL; Del Grosso, et al.^51^ and Schmer, et al.^53^ for Ithaca, NE; Del Grosso, et al.^51^ for Lafayette, IN, Clapp, et al.^54^ and Linden, et al.^55^ for Rosemount, MN.

## Supplementary information

Supplementary Information

## Data Availability

All data are publicly available in the cited references in this manuscript. Additionally, data are available from the corresponding author upon request. The code of the model used to conduct the watershed modeling is available at https://sites.google.com/view/tasc-model.

## Code availability

The code of the model used to conduct the watershed modeling is available at https://sites.google.com/view/tasc-model.
