# Supplementary material for: From basin to gulf: Conservation tillage improves soil health but exacerbates hypoxia
Source: NPJ Sustain Agric. 2025 Aug 28;3(1):47. doi: 10.1038/s44264-025-00090-0 (PMC12394069; doi:10.1038/s44264-025-00090-0)
Supplement: Supplementary file 1 — Supplementary Information [file 44264_2025_90_MOESM1_ESM.pdf]

Supplementary Information

From Basin to Gulf: Conservation Tillage Improves Soil Health but Exacerbates Hypoxia

Kang Liang, Xuesong Zhang, Gregory W. McCarty, Kaiguang Zhao, Feng Gao

Meta-analysis of soil organic carbon (SOC) and nitrate leaching

Supplementary Table 1. Reviewed literature for the meta-analysis on the impacts of tillage practices on SOC sequestration and nitrate leaching in U.S. corn-soybean production systems

Variable	Source	State	Latitude	Longitude
SOC	Edwards, et al. [1]	NE	34.30	-86.02
SOC	Hendrix, et al. [2]	GA	33.17	-84.33
SOC	Karlen, et al. [3]	IA	42.95	-92.54
SOC	Wander, et al. [4]	IL	39.78	-90.75
SOC	Lal [5]	OH	40.78	-81.95
SOC	Clapp, et al. [6]; Linden, et al. [7]	MN	44.71	-93.10
SOC	Al-Kaisi, et al. [8]	IA	42.93	-93.80
SOC	Jarecki and Lal [9]	OH	39.83	-83.63
SOC	Dolan, et al. [10]	MN	44.72	-93.10
SOC	Venterea, et al. [11]	MN	44.72	-93.10
SOC	Gál, et al. [12]	IN	40.47	-86.99
SOC	Huggins, et al. [13]	MN	44.07	-93.53
SOC	Mendoza, et al. [14]	NE	40.42	-96.79
SOC	Sainju, et al. [15]	AL	34.68	-86.87
SOC	Blanco-Canqui and Lal [16]	KY	38.22	-84.48
SOC	Jung, et al. [17]	MO	39.23	-92.12
SOC	Chatterjee and Lal [18]	MI	41.50	-81.70
SOC	Senthilkumar, et al. [19]	MI	42.40	-85.40
SOC	Ussiri and Lal [20]	OH	39.83	-83.63
SOC	Mishra, et al. [21]	OH	39.83	-83.63
SOC	Watts, et al. [22]	AL	34.29	-85.97
SOC	Jagadamma and Lal [23]	OH	40.78	-81.95
SOC	Varvel and Wilhelm [24]	NE	40.82	-96.70
SOC	Mirzaei, et al. [25]	IA	42.34	-93.42
SOC	Schmer, et al. [26,]Del Grosso, et al. [27]	NE	41.16	-96.41
SOC	Kumar, et al. [28]	MO	40.04	-83.05
SOC	Blanco-Canqui, et al. [29]	NE	42.49	-99.90
SOC	Devine, et al. [30]	GA	33.95	-83.38
SOC	Clay, et al. [31]	SD	44.30	-96.67
SOC	Alhameid, et al. [32]	SD	43.05	-96.89
SOC	Haruna and Nkongolo [33]	MO	38.97	-92.18
SOC	Blanco-Canqui, et al. [34]	NE	42.38	-96.98
SOC	Amorim, et al. [35]	MS	33.42	-90.92
Nitrate leaching	Karlen, et al. [3]	IA	43.16	-93.33
Nitrate leaching	Smith, et al. [36]	OK	34.89	-98.19
Nitrate leaching	Smith, et al. [36]	OK	35.58	-98.01
Nitrate leaching	Smith, et al. [36]	OK	36.40	-99.41
Nitrate leaching	McCracken, et al. [37]	ID	33.87	-83.42
Nitrate leaching	Logan, et al. [38]	OH	40.78	-81.93
Nitrate leaching	Randall and Iragavarapu [39]	ND	44.08	-93.51
Nitrate leaching	Meek, et al. [40]	ID	42.64	-111.81
Nitrate leaching	Singh and Kanwar [41]	IA	42.94	-92.57
Nitrate leaching	Bjorneberg, et al. [42]	IA	42.95	-92.55
Nitrate leaching	Weed and Kanwar [43]	IA	42.95	-92.55
Nitrate leaching	Tyler and Thomas [44]	MD	38.05	-84.54
Nitrate leaching	Kanwar, et al. [45]	IA	42.94	-92.57
Nitrate leaching	Bakhsh and Kanwar [46]	IA	42.94	-92.57
Nitrate leaching	Brye, et al. [47]	WI	43.28	-89.37
Nitrate leaching	Bakhsh, et al. [48]	IA	42.95	-92.55

Nitrate leaching	Masarik [49]	WI	43.28	-89.37
Nitrate leaching	Gupta, et al. [50]	WI	44.39	-89.60
Nitrate leaching	Stoddard, et al. [51]	KY	38.12	-84.50
Nitrate leaching	Endale, et al. [52]	GA	33.90	-83.40
Nitrate leaching	Masarik, et al. [53]	WI	43.28	-89.37
Nitrate leaching	Francesconi, et al. [54]	IN	41.46	-84.98
Nitrate leaching	Meisinger, et al. [55]	MD	39.02	-76.92
Nitrate leaching	Jabro, et al. [56]	ND	48.16	-103.10
Nitrate leaching	Waring, et al. [57]	IA	43.25	-94.83
Nitrate leaching	Dougherty, et al. [58]	IA	42.94	-92.57
Nitrate leaching	O'Brien, et al. [59]	IA	42.05	-93.71

Model evaluation for streamflow and water quality simulation

Supplementary Figure 1 illustrates the SWAT-C model (recently renamed to Terrestrial and Aquatic Sciences Convergence - TASC) simulation results of monthly streamflow (**Supplementary Figure 1(a)**), sediment load (**Supplementary Figure 1(b)**), nitrate load (**Supplementary Figure 1(c)**), and total nitrogen load (**Supplementary Figure 1(d)**) from the UMRB. The model performance was evaluated against observed data between 2006 and 2022. Overall, the model demonstrated its robustness in simulating both water quantity and quality. For example, the simulated streamflow closely matches with the observed flow data. The R^2, NSE, and PBIAS are 0.7, 0.73, and -1.8%, respectively. The model shows strong performance in simulating both TN and nitrate load, with R^2, NSE, and PBIAS values of 0.74, 0.65, and 1.9% for nitrate simulation, 0.79, 0.73, and 15.5% for TN, respectively. The model captured the trend of sediment load very well with the R^2 of 0.72, but modestly overestimated sediment load during high flow periods with the PBIAS of 32.4%.

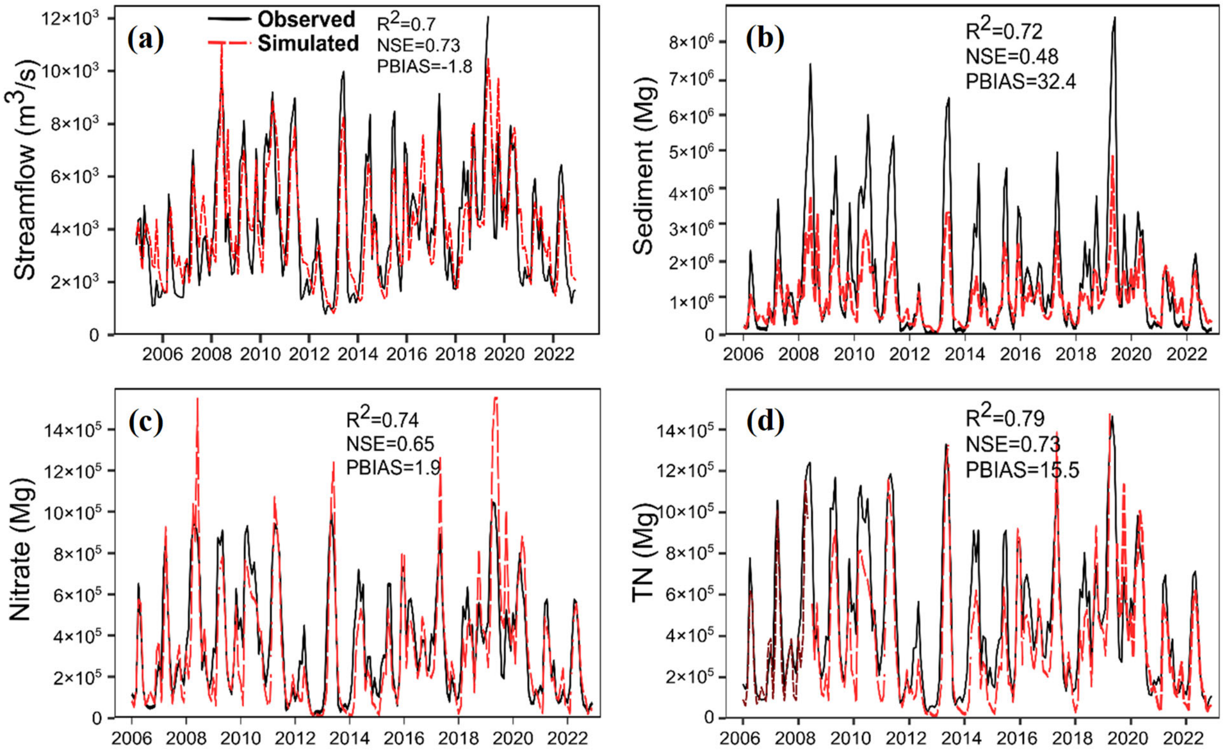

Supplementary Figure 1. SWAT-C/TASC performance evaluation. (a) streamflow, **(b)** sediment, **(c)** nitrate load, **(d)** total nitrogen load simulated by the SWAT-C/TASC model.

Model evaluation for simulating SOC under different tillage intensity

We assessed the performance of the SWAT-C/TASC model in simulating SOC. As shown in **Supplementary Figure 2(a)**, SWAT-C/TASC accurately captured the variation in SOC for the top 30 cm soil. The R^2, NSE, and PBIAS values were 0.95, 0.95, and 0.3%, respectively. The model also performed well for SOC simulation under different tillage practices, as demonstrated in **Supplementary Figure 2(b) - (d)**. For NT, the model achieved an R^2 of 0.95, NSE of 0.95, and PBIAS of 1.6%. Under IT, the corresponding values were R^2 = 0.98, NSE = 0.98, and PBIAS = 2.3%. For HT, the model achieved an R^2 of 0.78, NSE of 0.80, and PBIAS of 0.9%. Overall, the SWAT-C/TASC model demonstrated robust performance in simulating SOC under varied tillage practices.

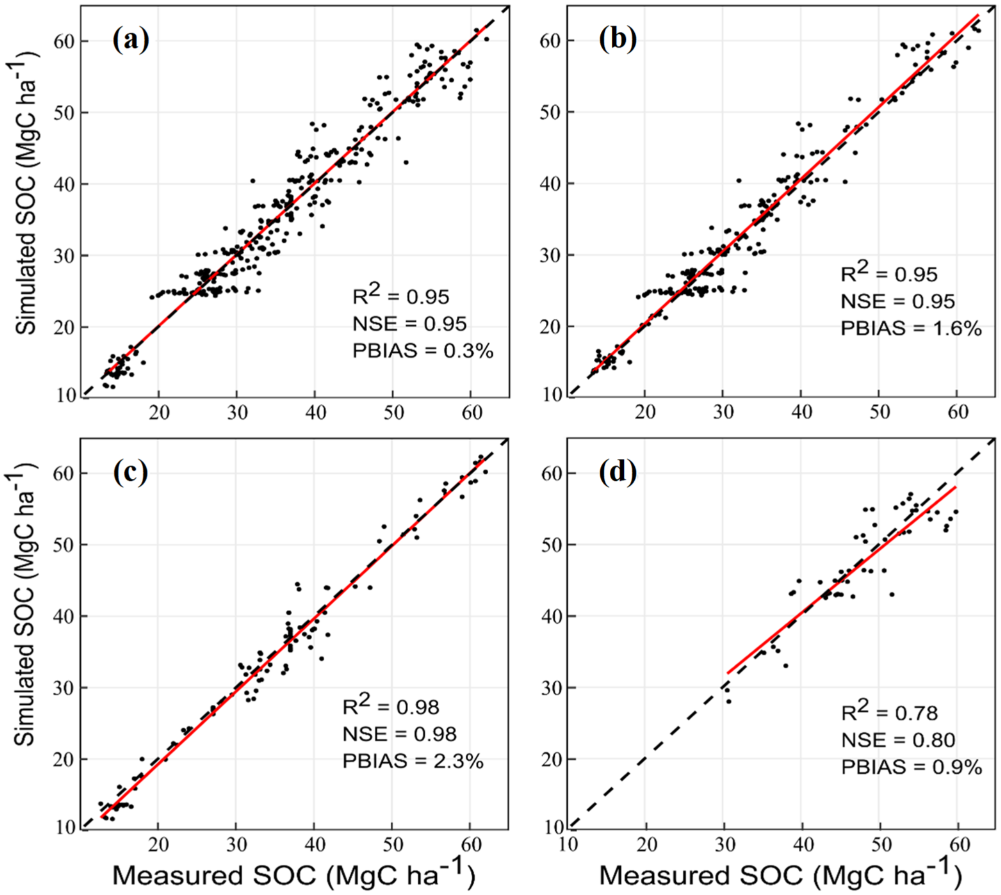

Supplementary Figure 2. SWAT-C/TASC performance evaluation for SOC simulation. (a) Top 30cm SOC, (**b)** SOC under no-till (NT), (**c)** SOC under intermediate intensity tillage (IT), (**d)** SOC under high intensity tillage (HT). The dashed line indicates the 1:1 line, while the solid red line represents the linear regression line.

REFERENCES

1 Edwards, J., Wood, C., Thurlow, D. & Ruf, M. Tillage and crop rotation effects on fertility status of a Hapludult soil. *Soil Science Society of America Journal* **56**, 1577-1582 (1992).

2 Hendrix, P. F., Franzluebbers, A. J. & McCracken, D. V. Management effects on C accumulation and loss in soils of the southern Appalachian Piedmont of Georgia. *Soil and Tillage Research* **47**, 245-251 (1998).

3 Karlen, D. L., Kumar, A., Kanwar, R. S., Cambardella, C. A. & Colvin, T. S. Tillage system effects on 15-year carbon-based and simulated N budgets in a tile-drained Iowa field. *Soil Tillage Res.* **48**, 155-165, doi:10.1016/s0167-1987(98)00142-1 (1998).

4 Wander, M., Bidart, M. & Aref, S. Tillage impacts on depth distribution of total and particulate organic matter in three Illinois soils. *Soil Science Society of America Journal* **62**, 1704-1711 (1998).
5 Lal, R. Long-term tillage and wheel traffic effects on soil quality for two central Ohio soils. *Journal of Sustainable Agriculture* **14**, 67-84 (1999).
6 Clapp, C. E., Allmaras, R. R., Layese, M. F., Linden, D. R. & Dowdy, R. H. Soil organic carbon and 13C abundance as related to tillage, crop residue, and nitrogen fertilization under continuous corn management in Minnesota. *Soil and Tillage Research* **55**, 127-142 (2000).
7 Linden, D. R., Clapp, C. E. & Dowdy, R. H. Long-term corn grain and stover yields as a function of tillage and residue removal in east central Minnesota. *Soil and Tillage Research* **56**, 167-174 (2000).
8 Al-Kaisi, M. M., Yin, X. & Licht, M. A. Soil carbon and nitrogen changes as affected by tillage system and crop biomass in a corn–soybean rotation. *Applied Soil Ecology* **30**, 174-191 (2005).
9 Jarecki, M. K. & Lal, R. Soil organic carbon sequestration rates in two long-term no-till experiments in Ohio. *Soil Science* **170**, 280-291 (2005).
10 Dolan, M. S., Clapp, C. E., Allmaras, R. R., Baker, J. M. & Molina, J. A. E. Soil organic carbon and nitrogen in a Minnesota soil as related to tillage, residue and nitrogen management. *Soil Tillage Res.* **89**, 221-231, doi:10.1016/j.still.2005.07.015 (2006).
11 Venterea, R. T., Baker, J. M., Dolan, M. S. & Spokas, K. A. Carbon and nitrogen storage are greater under biennial tillage in a Minnesota corn-soybean rotation. *Soil Science Society of America Journal* **70**, 1752-1762, doi:10.2136/sssaj2006.0010 (2006).
12 Gál, A., Vyn, T. J., Michéli, E., Kladivko, E. J. & McFee, W. W. Soil carbon and nitrogen accumulation with long-term no-till versus moldboard plowing overestimated with tilled-zone sampling depths. *Soil and Tillage Research* **96**, 42-51 (2007).
13 Huggins, D. R., Allmaras, R. R., Clapp, C. E., Lamb, J. A. & Randall, G. W. Corn-soybean sequence and tillage effects on soil carbon dynamics and storage. *Soil Science Society of America Journal* **71**, 145-154, doi:10.2136/sssaj2005.0231 (2007).
14 Mendoza, R., Franti, T., Doran, J. W., Powers, T. O. & Zanner, C. Tillage effects on soil quality indicators and nematode abundance in Loessial soil under long-term no-till production. *Communications in Soil Science and Plant Analysis* **39**, 2169-2190 (2008).
15 Sainju, U. M., Senwo, Z. N., Nyakatawa, E. Z., Tazisong, I. A. & Reddy, K. C. Tillage, cropping systems, and nitrogen fertilizer source effects on soil carbon sequestration and fractions. *Journal of Environmental Quality* **37**, 880-888 (2008).
16 Blanco-Canqui, H. & Lal, R. No-tillage and soil-profile carbon sequestration: An on-farm assessment. *Soil Science Society of America Journal* **72**, 693-701, doi:10.2136/sssaj2007.0233 (2008).
17 Jung, W. K., Kitchen, N. R., Sudduth, K. A. & Kremer, R. J. Contrasting grain crop and grassland management effects on soil quality properties for a north-central Missouri claypan soil landscape. *Soil science and plant nutrition* **54**, 960-971 (2008).
18 Chatterjee, A. & Lal, R. On farm assessment of tillage impact on soil carbon and associated soil quality parameters. *Soil Tillage Res.* **104**, 270-277, doi:10.1016/j.still.2009.03.006 (2009).
19 Senthilkumar, S., Kravchenko, A. & Robertson, G. Topography influences management system effects on total soil carbon and nitrogen. *Soil Science Society of America Journal* **73**, 2059-2067 (2009).
20 Ussiri, D. A. N. & Lal, R. Long-term tillage effects on soil carbon storage and carbon dioxide emissions in continuous corn cropping system from an alfisol in Ohio. *Soil Tillage Res.* **104**, 39-47, doi:10.1016/j.still.2008.11.008 (2009).
21 Mishra, U., Ussiri, D. A. N. & Lal, R. Tillage effects on soil organic carbon storage and dynamics in Corn Belt of Ohio USA. *Soil Tillage Res.* **107**, 88-96, doi:10.1016/j.still.2010.02.005 (2010).
22 Watts, D. B., Torbert, H. A., Prior, S. A. & Huluka, G. Long-Term Tillage and Poultry Litter Impacts Soil Carbon and Nitrogen Mineralization and Fertility. *Soil Science Society of America Journal* **74**, 1239-1247, doi:10.2136/sssaj2008.0415 (2010).
23 Jagadamma, S. & Lal, R. Distribution of organic carbon in physical fractions of soils as affected by agricultural management. *Biology and Fertility of Soils* **46**, 543-554 (2010).

24 Varvel, G. E. & Wilhelm, W. W. Long-Term Soil Organic Carbon as Affected by Tillage and Cropping Systems. *Soil Science Society of America Journal* **74**, 915-921, doi:10.2136/sssaj2009.0362 (2010).
25 Mirzaei, M. *et al.* Crop residues in corn-wheat rotation in a semi-arid region increase CO₂ efflux under conventional tillage but not in a no-tillage system. *Pedobiologia* **93-94**, 11, doi:10.1016/j.pedobi.2022.150819 (2022).
26 Schmer, M. R., Jin, V. L., Wienhold, B. J., Varvel, G. E. & Follett, R. F. Tillage and Residue Management Effects on Soil Carbon and Nitrogen Under Irrigated Continuous Corn. *Soil Science Society of America Journal* **78**, 1987-1996, doi:10.2136/sssaj2014.04.0166 (2014).
27 Del Grosso, S. J. *et al.* Introducing the GRACEnet/REAP Data Contribution, Discovery, and Retrieval System. *Journal of Environmental Quality* **42**, 1274-1280, doi:10.2134/jeq2013.03.0097 (2013).
28 Kumar, S. *et al.* Long-term tillage and drainage influences on soil organic carbon dynamics, aggregate stability and corn yield. *Soil Science and Plant Nutrition* **60**, 108-118, doi:10.1080/00380768.2013.878643 (2014).
29 Blanco-Canqui, H., Ferguson, R. B., Shapiro, C. A., Drijber, R. A. & Walters, D. T. Does inorganic nitrogen fertilization improve soil aggregation? Insights from two long-term tillage experiments. *Journal of environmental quality* **43**, 995-1003 (2014).
30 Devine, S., Markewitz, D., Hendrix, P. & Coleman, D. Soil aggregates and associated organic matter under conventional tillage, no-tillage, and forest succession after three decades. *PloS one* **9**, e84988 (2014).
31 Clay, D. E. *et al.* Tillage and corn residue harvesting impact surface and subsurface carbon sequestration. *J Environ Qual* **44**, 803-809, doi:10.2134/jeq2014.07.0322 (2015).
32 Alhameid, A., Ibrahim, M., Kumar, S., Sexton, P. & Schumacher, T. E. Soil Organic Carbon Changes Impacted by Crop Rotational Diversity under No-Till Farming in South Dakota, USA. *Soil Science Society of America Journal* **81**, 868-877, doi:10.2136/sssaj2016.04.0121 (2017).
33 Haruna, S. & Nkongolo, N. Tillage, Cover Crop and Crop Rotation Effects on Selected Soil Chemical Properties. *Sustainability* **11**, doi:10.3390/su11102770 (2019).
34 Blanco-Canqui, H., Shapiro, C., Jasa, P. & Iqbal, J. No-till and carbon stocks: Is deep soil sampling necessary? Insights from long-term experiments. *Soil Tillage Res.* **206**, doi:10.1016/j.still.2020.104840 (2021).
35 Amorim, H. C. S. *et al.* No-till impacts on soil organic carbon and soil quality in the Lower Mississippi River basin: Implications for sustainable management. *Soil Science Society of America Journal*, doi:10.1002/saj2.20717 (2024).
36 Smith, S., Sharpley, A., Naney, J., Berg, W. & Jones, O. Water quality impacts associated with wheat culture in the Southern Plains. Report No. 0047-2425, (Wiley Online Library, 1991).
37 McCracken, D. *et al.* in *1993 Southern Conservation Tillage Conference for Sustainable Agriculture.* 11 (Citeseer).
38 Logan, T., Eckert, D. & Beak, D. Tillage, crop and climatic effects of runoff and tile drainage losses of nitrate and four herbicides. *Soil and Tillage Research* **30**, 75-103 (1994).
39 Randall, G. & Iragavarapu, T. Impact of long-term tillage systems for continuous corn on nitrate leaching to tile drainage. Report No. 0047-2425, (Wiley Online Library, 1995).
40 Meek, B., Carter, D., Westermann, D., Wright, J. & Peckenpaugh, R. Nitrate leaching under furrow irrigation as affected by crop sequence and tillage. *Soil Science Society of America Journal* **59**, 204-210 (1995).
41 Singh, P. & Kanwar, R. S. Simulating NO3-N transport to subsurface drain flows as affected by tillage under continuous corn using modified RZWQM. *Transactions of the ASAE* **38**, 499-506 (1995).
42 Bjorneberg, D. L., Kanwar, R. S. & Melvin, S. W. Seasonal changes in flow and nitrate-N loss from subsurface drains. *Transactions of the ASAE* **39**, 961-967 (1996).
43 Weed, D. & Kanwar, R. Nitrate and water present in and flowing from root-zone soil. Report No. 0047-2425, (Wiley Online Library, 1996).
44 Tyler, D. D. & Thomas, G. W. Lysimeter measurements of nitrate and chloride losses from soil under conventional and no-tillage corn. Report No. 0047-2425, (Wiley Online Library, 1977).

45 Kanwar, R. S., Colvin, T. S. & Karlen, D. L. Ridge, moldboard, chisel, and no-till effects on tile water quality beneath two cropping systems. *Journal of Production Agriculture* **10**, 227-234 (1997).
46 Bakhsh, A. & Kanwar, R. S. Simulating tillage effects on nonpoint source pollution from agricultural lands using GLEAMS. *Transactions of the ASAE* **44**, 891 (2001).
47 Brye, K., Norman, J., Bundy, L. & Gower, S. Nitrogen and carbon leaching in agroecosystems and their role in denitrification potential. *Journal of Environmental Quality* **30**, 58-70 (2001).
48 Bakhsh, A. *et al.* Cropping system effects on NO3–N loss with subsurface drainage water. *Transactions of the ASAE* **45**, 1789 (2002).
49 Masarik, K. C. *Monitoring water drainage and nitrate leaching below different tillage practices and fertilization rates*, University of Wisconsin--Madison, (2003).
50 Gupta, S., Munyankusi, E., Moncrief, J., Zvomuya, F. & Hanewall, M. Tillage and manure application effects on mineral nitrogen leaching from seasonally frozen soils. *Journal of environmental quality* **33**, 1238-1246 (2004).
51 Stoddard, C., Grove, J. H., Coyne, M. S. & Thom, W. O. Fertilizer, tillage, and dairy manure contributions to nitrate and herbicide leaching. *Journal of Environmental Quality* **34**, 1354-1362 (2005).
52 Endale, D. M., Schomberg, H. H., Jenkins, M. B., Franklin, D. H. & Fisher, D. S. Management implications of conservation tillage and poultry litter use for Southern Piedmont USA cropping systems. *Nutr. Cycl. Agroecosyst.* **88**, 299-313, doi:10.1007/s10705-009-9318-z (2010).
53 Masarik, K. C., Norman, J. M. & Brye, K. R. Long-term drainage and nitrate leaching below well-drained continuous corn agroecosystems and a prairie. *Journal of Environmental Protection* **2014** (2014).
54 Francesconi, W., Smith, D. R., Heathman, G. C., Wang, X. & Williams, C. O. Monitoring and APEX modeling of no-till and reduced-till in tile-drained agricultural landscapes for water quality. *Transactions of the ASABE* **57**, 777-789 (2014).
55 Meisinger, J. J., Palmer, R. E. & Timlin, D. J. Effects of tillage practices on drainage and nitrate leaching from winter wheat in the Northern Atlantic Coastal-Plain USA. *Soil and Tillage Research* **151**, 18-27 (2015).
56 Jabro, J. D., Iversen, W. M., Stevens, W. B., Sainju, U. M. & Allen, B. L. Tillage effects on drainage fluxes and nitrate leaching through unsaturated zone under irrigated corn-soybean rotation. *Applied Engineering in Agriculture* **35**, 293-300 (2019).
57 Waring, E. R., Pederson, C., Lagzdins, A., Clifford, C. & Helmers, M. J. Water and soil quality respond to no-tillage and cover crops differently through 10 years of implementation. *Agric. Ecosyst. Environ.* **360**, 11, doi:10.1016/j.agee.2023.108791 (2024).
58 Dougherty, B. W. *et al.* Midwestern cropping system effects on drainage water quality and crop yields. Report No. 0047-2425, (Wiley Online Library, 2020).
59 O'Brien, P. L. *et al.* Nitrate losses and nitrous oxide emissions under contrasting tillage and cover crop management. Report No. 0047-2425, (Wiley Online Library, 2022).
